# A Non-targeted Metabolomics Approach Unravels the VOCs Associated with the Tomato Immune Response against *Pseudomonas syringae*

**DOI:** 10.3389/fpls.2017.01188

**Published:** 2017-07-04

**Authors:** María Pilar López-Gresa, Purificación Lisón, Laura Campos, Ismael Rodrigo, José Luis Rambla, Antonio Granell, Vicente Conejero, José María Bellés

**Affiliations:** Instituto de Biología Molecular y Celular de Plantas, Universitat Politècnica de València – Consejo Superior de Investigaciones Científicas, Ciudad Politécnica de la InnovaciónValencia, Spain

**Keywords:** metabolomics, tomato, bacteria, VOCs, defense

## Abstract

Volatile organic compounds (VOCs) emitted by plants are secondary metabolites that mediate the plant interaction with pathogens and herbivores. These compounds may perform direct defensive functions, i.e., acting as antioxidant, antibacterial, or antifungal agents, or indirectly by signaling the activation of the plant’s defensive responses. Using a non-targeted GC-MS metabolomics approach, we identified the profile of the VOCs associated with the differential immune response of the Rio Grande tomato leaves infected with either virulent or avirulent strains of *Pseudomonas syringae* DC3000 pv. *tomato*. The VOC profile of the tomato leaves infected with avirulent bacteria is characterized by esters of (Z)-3-hexenol with acetic, propionic, isobutyric or butyric acids, and several hydroxylated monoterpenes, e.g., linalool, α-terpineol, and 4-terpineol, which defines the profile of an immunized plant response. In contrast, the same tomato cultivar infected with the virulent bacteria strain produced a VOC profile characterized by monoterpenes and SA derivatives. Interestingly, the differential VOCs emission correlated statistically with the induction of the genes involved in their biosynthetic pathway. Our results extend plant defense system knowledge and suggest the possibility for generating plants engineered to over-produce these VOCs as a complementary strategy for resistance.

## Introduction

Plants have developed multiple defense mechanisms to protect themselves against biotic and abiotic stresses. Accumulation of secondary metabolites, which display numerous biological properties, constitutes a major component of stress responses (Dixon, 2001; Pusztahelyi et al., 2015; Qian et al., 2015; Kalaivani et al., 2016). Among them, volatile organic compounds (VOCs) are a relevant group involved in plant protection against pathogens and herbivores, and also in attracting pollinators and seed dispersers (Dudareva et al., 2013). Plant VOCs include a wide range of chemical structures, such as terpenoids and phenylpropanoids-benzenoids, as well as fatty and amino acid derivatives (Holopainen and Gershenzon, 2010; Granell and Rambla, 2013). They all have a low molecular weight and polarity, high vapor pressures, and possess the ability to both cross membranes freely and be released into the surrounding atmosphere (Dudareva et al., 2006).

The bacterial speck of tomato, caused by *Pseudomonas syringae* pv. *tomato* (*Pst*), is a major problem in the agricultural industry (Willis and Kinscherf, 2009). Tomato cultivar Rio Grande, which contains the *Pto* gene (RG-*Pto*), is resistant to *Pst*, which expresses effectors or avirulence genes *avrPto/avrPtoB*. Such “gene-for-gene” recognition (*Pto-avrPto/avrPtoB*) elicits Effector-Triggered Immunity (ETI) establishment in plants, which allows the control of bacterial spread, and results in an incompatible interaction (Jia et al., 1997; Dangl and Jones, 2001; Lin and Martin, 2005). In contrast, *Pst*, which bears a deletion of *avrPto* genes (Δ*avrPto*/Δ*avrPtoB*), becomes virulent to RG-*Pto* plants by causing disease in plants and the development of a compatible interaction (Salmeron et al., 1994). Therefore, this tomato pathosystem represents an excellent model to study both kinds of plant–pathogen interactions.

Different signal molecules, such as salicylic acid (SA), gentisic acid (GA), ethylene (ET), or jasmonic acid (JA), have been described to accumulate upon pathogen attacks in tomato plants. SA accumulation has been associated with avirulent infections (Block et al., 2005), while high levels of GA and ET have been found in compatible interactions in tomato (Bellés et al., 1999; Zacarés et al., 2007). JA has been associated with plant responses to herbivores or necrotrophic pathogens (Zhang et al., 2017). However, the role of these defensive molecules has not been characterized in tomato Rio Grande plants infected with virulent or avirulent *Pst* strains.

More and more studies are being conducted to understand the participation of small metabolites in plant–pathogen interactions (Allwood et al., 2008, 2012). However, the interest in VOCs has focused mainly on the plant response to herbivores and fruit quality, and studies of VOCs in the plant response to pathogens are scarce (Niinemets et al., 2013). Specifically, differential volatile emission has been described for tobacco and pepper leaves in response to both avirulent and virulent strains of *Pseudomonas* (Huang et al., 2003, 2005) and *Xanthomonas* (Cardoza and Tumlinson, 2006), respectively, but the biological meaning of this phenomenon is not well understood.

In this paper, we applied an untargeted GC-MS metabolomics approach to analyze the volatiles differentially emitted in RG-*Pto* tomato plants infected with either avirulent strain *Pst* DC3000 or virulent strain *Pst* DC3000 Δ*avrPto*/Δ*avrPtoB*. Besides, levels of classical defense hormones, such as SA, GA, ET, and JA, were characterized in both tomato interactions. Finally, the activation of a set of genes involved in the corresponding VOC biosynthesis pathways was also studied. Our results will unravel the VOC network that underlies the tomato immune response against *Pseudomonas syringae*.

## Materials and Methods

### Bacterial Strains, Growth Conditions, and Inoculum Preparation

The bacterial strains used in this study were *Pseudomonas syringae* pv. *tomato* DC3000 (*Pst* DC3000), and *Pst* DC3000 that contains deletions in genes *avrPto* and *avrPtoB* (*Pst* DC3000 Δ*avrPto*/Δ*avrPtoB*) (Ntoukakis et al., 2009). Bacteria were grown overnight at 28°C in 20 mL Petri dishes with King’s B agar medium supplemented with different antibiotic doses: rifampicin (10 mg/mL) and kanamicin (0.5 mg/mL) for *Pst* DC3000, and rifampicin (10 mg/mL), kanamycin (0.25 mg/mL) and spectinomycin (2.5 mg/mL) for *Pst* DC3000 *ΔavrPto/ΔavrPtoB*. Then bacterial colonies were transferred to 15 mL of King’s B medium supplemented with rifampicin (10 mg/mL), and were grown overnight at 28°C with stirring. Bacteria were then pelleted by centrifugation and resuspended in 10 mM of MgCl_2_, which contained 0.05% (v/v) Silwet L-77, to an optical density of 0.1 at 600 nm. Dilution plating was used to determine the final inoculum concentration, which averaged at 1 × 10^7^ CFU/mL.

### Plant Material and Bacterial Inoculation

Tomato seeds from the cultivar Rio Grande that contained resistance (*R*) gene *Pto* (RG-*Pto*) were grown under greenhouse conditions with a 16/8-h (26/30°C) light/dark photoperiod (300 μmol/m^2^/s) and 65% relative humidity in 12 cm-diameter pots that contained a 1:1 mixture of peat and vermiculite.

Inoculations with compatible and incompatible bacteria were produced by immersing 28-day-old RG-*Pto* plants in *Pst* DC3000 or *Pst* DC3000 Δ*avrPto*/Δ*avrPtoB* suspension, respectively, as previously described (Martin et al., 1993). For mock inoculations, plants were dipped in 10 mM of MgCl_2_ solution that contained Silwet L-77 (0.05%) without the bacterial inoculum. The third and fourth leaves, from bottom to top, were harvested and frozen in liquid nitrogen at the indicated times. The fifth leaf was placed freshly in a 10-mL screw cap vial and was kept for 5 h to take ET measurements. Six biological replicates were analyzed for each time and tomato–bacteria interaction.

### Extraction and the HPLC Analysis of Salicylic and Gentisic Acids

Extraction of free and total SA and GA from tomato leaflets was performed according to our previously published protocol (Bellés et al., 2008). Aliquots of 30 μL were injected through a Waters 717 autosampler into a reverse-phase Sun Fire 5-μm C18 column (4.6 mm ×150 mm) equilibrated in 1% (v/v) acetic acid at room temperature. A 20-min linear gradient of 1% acetic acid to 100% methanol was applied using a 1525 Waters Binary HPLC pump at a flow rate of 1 mL/min. SA and GA were detected with a 2475 Waters Multi-λ Fluorescence detector (λ excitation 313 nm; λ emission 405 nm), and were quantified with the Waters Empower Pro software using authentic standard compounds (SA sodium salt and GA, Sigma–Aldrich, Madrid, Spain). Standard curves were performed for each compound using similar concentration ranges to those detected in the samples. Data were corrected for losses in the extraction procedure, and recovery of metabolites ranged between 50 and 80%.

### Jasmonic Acid and Ethylene Measurements

Ethylene production was measured as described by Lieberherr et al. (2003) with some modifications. Approximately 0.5 g of fresh tissue from the fifth tomato leaf, harvested at the indicated time points after bacterial inoculation, was quickly enclosed in gas-tight 10-mL glass vials fitted with a septum. After 5 h, 400 μL of the gas phase from the vial were analyzed by gas chromatography in a 4890A Hewlett Packard gas chromatograph fitted with a flame ionization detector (FID) using a Teknokroma capillary column (2 m × 1/6″ OD × 1 mm ID, Alumina F1 80/100). The carrier gas was helium, used at a pre-column pressure of 140 kPa. The injector and detector temperatures were set at 200°C, while the oven temperature was 80°C. The retention time of the ET peak under these conditions was 2.5 min. For each time point, six replicates were analyzed and the amount of ET was calculated from the data recorded and analyzed with the MassLynx Waters software by constructing a standard ET curve.

For JA quantification, 250 mg of frozen tissue from the third and fourth tomato leaves were added to 80% methanol–1% acetic acid that contained the internal standard dihydrojasmonate (OlChemIm, Czechia), and were mixed by shaking for 1 h at 4°C. The extract was kept at -20°C overnight and was then centrifuged. The supernatant was dried in a vacuum evaporator. The dry residue was dissolved in 1% acetic acid and passed through a reverse phase Oasis HLB column (Seo et al., 2011). The dried eluate was dissolved in 5% acetonitrile–1% acetic acid, and the hormone was identified using a reverse phase Ultra Performance Liquid Chromatography (UPLC) system coupled to a Q-Exactive mass spectrometer (Orbitrap detector; Thermo Fisher Scientific) by targeted Selected Ion Monitoring (SIM). Separation was performed in a 2.6 μm Accucore RP-MS column, 50 mm long × 2.1 mm i.d.; (Thermo Fisher Scientific) using a 5–50% acetonitrile gradient that contained 0.05% acetic acid as the solvent system at 400 μL/min for 14 min. The JA concentration in the extracts was determined using embedded calibration curves with the authentic standard (OlChemIm, Czechia) and the Xcalibur 2.2 SP1 build 48 and TraceFinder software.

### HS-SPME Extraction and the GC-MS Analysis of Volatile Compounds

For the volatile analysis, 100 mg of frozen tomato leaf powder were weighed in a 10-mL headspace screw-cap vial. One milliliter of a saturated CaCl_2_ solution and 100 μL of 750 mM EDTA (adjusted to 7.5 pH with NaOH) were added, mixed gently and sonicated for 5 min. Extraction of volatile compounds was performed by headspace solid-phase micro-extraction (HS-SPME) (Rambla et al., 2015). The pre-incubation and extraction periods, both at 50°C, were 10 and 20 min, respectively. Adsorption was performed by means of a 65 μm DVB/PDMS fiber (Supelco, Bellefonte, PA, United States). Desorption was done in the injection port of the gas chromatograph for 1 min at 250°C in the splitless mode. Volatile extraction and injection were performed automatically with a CombiPAL autosampler (CTC Analytics, Zwingen, Switzerland).

The chromatographic separation of compounds was performed in an Agilent 6890N gas chromatograph (Santa Clara, CA, United States) equipped with a DB-5 ms fused silica capillary column (60 m long, 0.25 mm i.d., 1 μm film thickness). The oven temperature conditions were 40°C for 2 min, 5°C/min ramp until 250°C and then held isothermally at 250°C for 5 min. Helium was used as the carrier gas at 1.2 mL/min at a constant flow. Detection was performed in an Agilent 5975B mass spectrometer (Santa Clara, CA, United States), which operated in the EI mode (ionization energy, 70 eV; source temperature 230°C). Data acquisition was performed in the scan mode (*m/z* range 35–250; six scans per second). Chromatograms and mass spectra were recorded and processed by the Enhanced ChemStation software (Agilent).

The unequivocal compound identification of the 70 volatile compounds was carried out by comparing both mass spectra and retention times with those of pure standards. All the commercial standards were purchased from Sigma–Aldrich (Madrid, Spain). Three other compounds were tentatively identified by comparing their mass spectra with those in the NIST 05 Mass Spectral library. Such tentatively identified compounds are marked with an asterisk.

### RNA Extraction and the Quantitative RT-PCR Analysis

The total RNA of tomato leaves was extracted using TRIzol reagent (Invitrogen, Carlsbad, CA, United States), following the manufacturer’s protocol. RNA was then precipitated by adding one volume of 6 M LiCl and keeping it on ice for 4 h. Afterward the pellet was washed using 3 M LiCl and was dissolved in RNase-free water. Finally, in order to remove any contaminating genomic DNA, 2 U of TURBO DNase (Ambion, Austin, TX, United States) were added per microliter of RNA.

For the quantitative RT-PCR (qRT-PCR) analysis, one microgram of total RNA was employed to obtain the corresponding cDNA target sequences using an oligo(dT)_18_ primer and the PrimeScript RT reagent kit (Perfect Real Time, Takara Bio Inc., Otsu, Shiga, Japan), following the manufacturer’s directions. Quantitative PCR was carried out as previously described (Campos et al., 2014). A housekeeping gene transcript, *Elongation Factor 1 alpha* (eEF1α), was used as the endogenous reference. The PCR primers were designed using the pcr Efficiency software (Mallona et al., 2011) and are listed in Supplementary Table S1. The primers used to amplify *TomLOXF* have been previously described (Shen et al., 2014).

### Statistical Analysis

The statistical analyses of the signal compounds levels (GA, SA, ET, and JA) and the qRT-PCR of the selected genes were done by an analysis of variance (multifactor ANOVA) using Statgraphics Centurion XVI.

For the untargeted analysis of the volatile profile, the GC-MS data were processed with the MetAlign software (Wageningen, Netherlands) for the alignment of the chromatograms and the quantitation of each MS feature. The resulting dataset was submitted to a Partial Least Square (PLS) study by the SIMCA-P software (v. 11.0, Umetrics, Umeå, Sweden) using unit variance (UV) scaling.

For the Hierarchical Cluster Analysis (HCA), the ratios for each VOC were calculated and log_2_-transformed for normalization. HCA was performed with the Acuity 4.0 software (Axon Instruments) with the distance metrics based on the Pearson correlation. The normalized data were represented as a heat map using the same software. The Pearson correlations between gene expression and the VOCs concentrations were performed with the SPSS 16.0 software by considering data at 10 and 18 hpi (hour post-inoculation).

## Results

### RG-*Pto* Tomato Plants Infected Either with the Avirulent or Virulent Bacterial Strain Displayed Noticeable Symptom Differences

**FIGURE 1:**
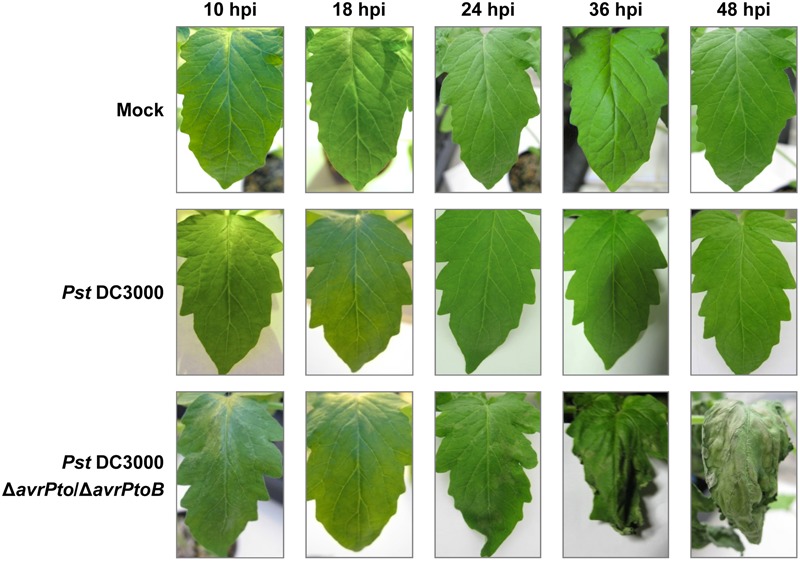
Symptom evolution in RG-*Pto* plants at 10, 18, 34, 36, and 48 h post-inoculation (hpi) with 10 mM of MgCl_2_ solution (mock), or either *Pst* DC3000 or *Pst* DC3000 Δ*avrPto*/Δ*avrPtoB* at 10^7^ CFU/mL.

Symptoms development of the tomato plants infected with either avirulent strain *Pst* DC3000 or virulent *Pst* DC3000 Δ*avrPto*/Δ*avrPtoB* is shown in **Figure 1**. These symptoms ranged on a 0–4 scale as follows: symptomless (0), weak (1), moderate (2), severe (3), and very severe (4). Inoculation of the RG-*Pto* tomato plants with the virulent strain resulted in chlorotic lesions appearing by 18 hpi, which displayed a symptom degree from (0) to (1). These initial lesions increased in intensity and size, and caused significant leaf damage that ranged from (2) to (3) at 24 and 36 hpi, respectively. By 48 hpi, necrotic lesions extended to the total area, and leaves lost their firmness and completely collapsed, which was the maximum level of symptomatology (4). Strong epinasty was observed in the leaves of these symptomatic tomato plants. In contrast, no symptoms (0) were observed at any time on the RG-*Pto* tomato plants inoculated with avirulent strain *Pst* DC3000 due to *Pto-avrPto/avrPtoB* recognition and ETI establishment. Therefore, these immunized tomato leaves were similar to the mock-inoculated plants.

### Levels of Salicylic Acid, Gentisic Acid, and Ethylene Were Enhanced in the RG-*Pto* Tomato Plants Infected with the Virulent Bacterial Strain, While Jasmonic Acid Drastically Lowered

**FIGURE 2:**
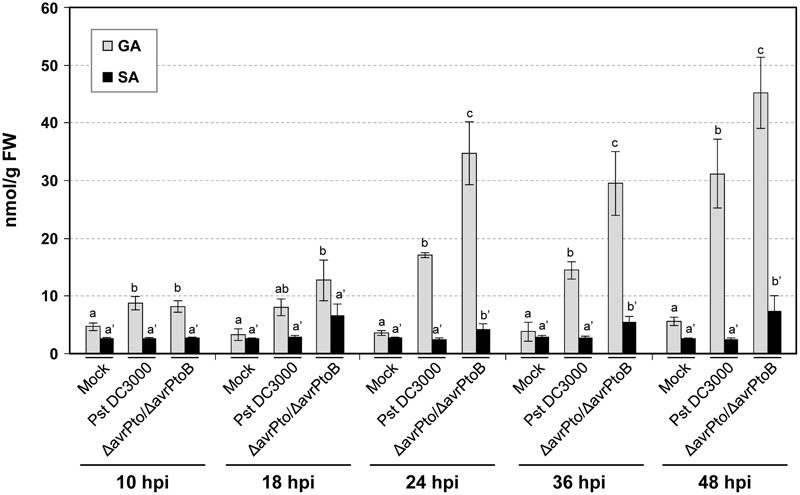
Levels of the free and total salicylic (SA) and gentisic (GA) acids, measured in an HPLC-fluorescence detector in the mock-inoculated RG-*Pto* tomato plants (mock) and upon infection with *Pst* DC3000 or *Pst* DC3000 Δ*avrPto*/Δ*avrPtoB* at 10, 18, 24, 36, and 48 hpi. Two multifactor ANOVA analyses were performed using GA (x) or SA (x’) data. Different letters indicate the statistical significances with a *p-*value < 0.05 compared with the mock-inoculated plants.

The levels of the signaling defense molecules, SA, GA, ET, and JA were analyzed in the plants infected with both virulent and avirulent strains in a time-course study. **Figure 2** shows the significant GA induction that occurred in all the inoculated plants that bore both interactions. The increased amount of GA was already evident in tomato plants at 10 hpi. Leaf GA accumulation increased as bacterial infection progressed, and the highest levels peaked at 48 hpi (45 nmol/g FW) in the symptomatic tomato plant leaves. By 24 hpi, a significantly higher GA value was obtained in the compatible interaction compared to the incompatible infection. No remarkable increments in SA were observed in the *Pseudomonas*-infected tomato plants at any time point, although differences in the SA levels started to become significant in the compatible interaction by 24 hpi.

**FIGURE 3:**
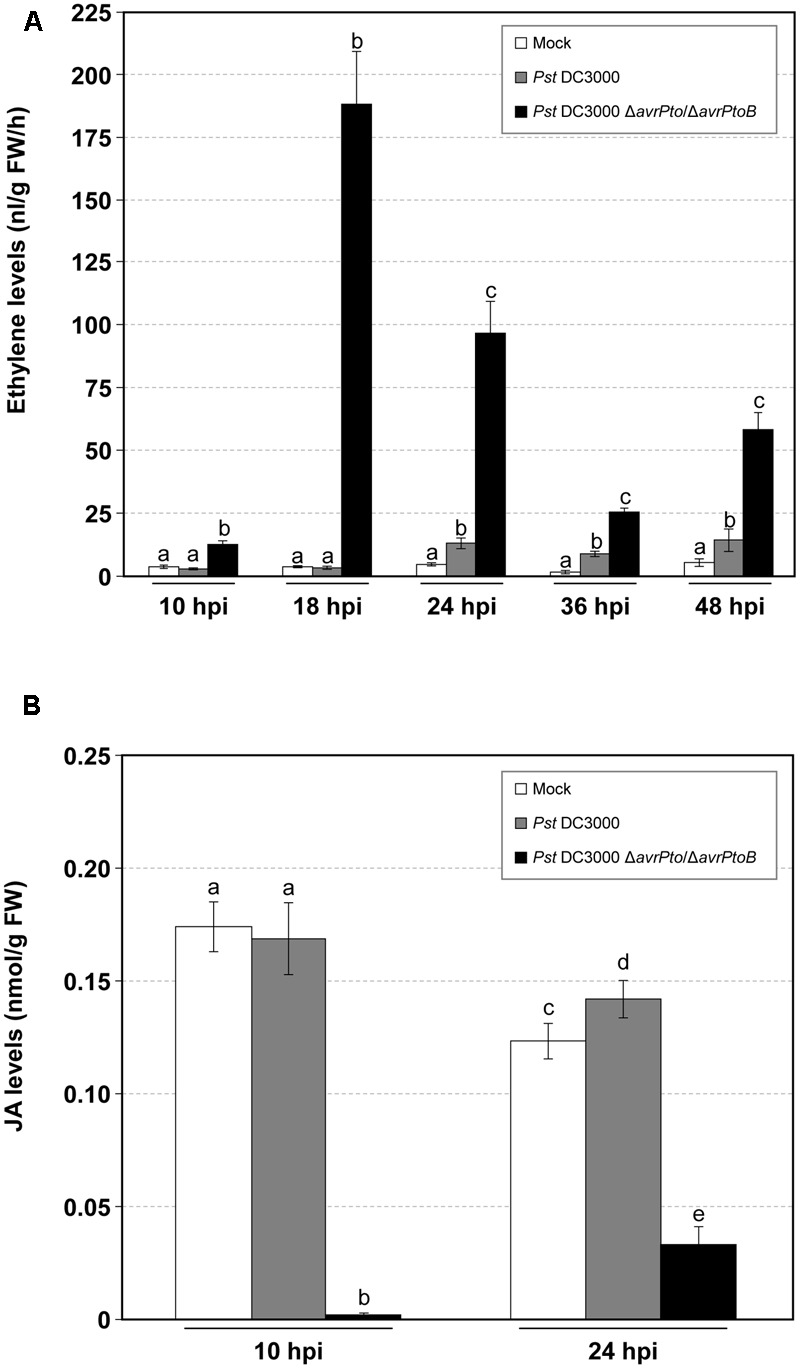
Time-course analysis of the mock-inoculated RG-*Pto* tomato plants (mock) and upon infection with *Pst* DC3000 or *Pst* DC3000 Δ*avrPto*/Δ*avrPtoB* at 10, 18, 24, 36, and 48 hpi for ethylene production **(A)**, and at 10 and 24 hpi for jasmonic acid (JA) accumulation **(B)**. Different letters indicate the statistical significances with a *p-*value < 0.05 compared with the mock-inoculated plants.

The evolution of ET from the leaves of both the compatible and incompatible tomato interactions was also measured. A dramatic increase in the production of this stress hormone was detected in the compatible interaction, which paralleled GA accumulation and appearance of symptoms (**Figure 3A**). Maximum ET production (188 nL/gFW/h) was reached at 18 hpi, which coincided with the onset of symptom development. These high levels of ET (up to 10-fold) could explain the strong epinasty observed in the tomato plants infected with the virulent bacteria. Remarkably during this compatible infection, ET biosynthesis was higher than that observed in the incompatible interaction, and was significantly elevated at any time point. Regarding infection with the avirulent bacteria, the differences in ET emission between the infected and mock-inoculated plants was only significant at 24, 36, and 48 hpi.

In contrast, the JA levels drastically lowered during virulent infection, and almost non-detectable values were displayed at 10 hpi. Regarding avirulent infection, JA production remained unaltered compared to the mock-inoculated tomato plants. A similar tendency was observed at 24 hpi (**Figure 3B**).

### The Volatile Profile of Tomato Leaves Altered Differentially upon Infection with Both *Pseudomonas syringae* Strains

In order to examine the VOCs involved in the tomato–pathogen interactions, changes in the levels of these metabolites in the RG-*Pto* tomato plants, which were either mock-inoculated or infected with avirulent strain *Pst* DC3000 or virulent *Pst* DC3000 Δ*avrPto*/Δ*avrPtoB*, were analyzed by GC-MS in the leaves infected between 10 and 48 hpi (see Materials and Methods). **Table 1** lists the VOCs detected on our HS-SPME/GC-MS platform for the mock and infected RG-*Pto* tomato plants. In all, 73 compounds were identified: 11 esters (9 aliphatic and 2 aromatic), 20 aldehydes (16 aliphatic, 3 aromatic and 1 norcarotenoid), 13 alcohols (6 aliphatic, 1 norcarotenoid, 5 monoterpenic, and 1 sesquiterpenic), 9 monoterpene hydrocarbons, 8 ketones (5 aliphatic, 1 aromatic, and 2 norcarotenoid), 3 sesquiterpene hydrocarbons, 2 furans, 2 nitriles, 4 aliphatic acids, and 1 aromatic hydrocarbon. They were all unequivocally confirmed by using pure standards, except for three of them, which were tentatively identified based on their mass spectra similarity (match > 900).

**Table 1:** List of the VOCs identified in tomato leaves by GC-MS.

	Volatile Organic	Family	Retention	Specific
Code	Compound	Code/Number	time (min)	ion (m/z)
1	Ethanol	Alc/1	4.95	45
2	Acetone	Ket/1	5.68	58
3	Butanol	Alc/2	10.41	56
4	1-Penten-3-ol	Alc/3	11.17	57
5	1-Penten-3-one	Ket/2	11.29	55
6	2-Pentanone	Ket/3	11.31	86
7	3-Pentanone	Ket/4	11.68	86
8	Pentanal	Ald/1	11.79	44
9	2-Ethylfuran	Fur/1	11.85	81
10	3-Methylbutanenitrile	Nit/1	13.38	43
11	(*E*)-2-Methyl-2-butenal	Ald/2	13.62	84
12	(*E*)-2-Pentenal	Ald/3	14.07	83
13	1-Pentanol	Alc/4	14.36	42
14	(*Z*)-2-Penten-1-ol	Alc/5	14.48	68
15	(*Z*)-3-Hexenal	Ald/4	15.75	69
16	Hexanal	Ald/5	15.84	72
17	Butyl acetate	Est/1	16.18	43
18	Methyl pentanoate	Est/2	16.65	85
19	(*Z*)-3-Hexen-1-ol	Alc/6	18.01	82
20	(*E*)-2-Hexenal	Ald/6	18.03	83
21	Pentanoic acid	Acid/1	18.29	60
22	2-Heptanone	Ket/5	19.30	58
23	Heptanal	Ald/7	19.85	70
24	Methyl hexanoate	Est/3	20.47	74
25	(*E,E*)-2,4-Hexadienal	Ald/8	20.35	81
26	α-Pinene	Mt hd/1	21.51	93
27	Hexanoic acid	Acid/2	21.95	60
28	(*E*)-2-Heptenal	Ald/9	22.02	68
29	Benzaldehyde	Ald/10	22.66	106
30	*o*-Cymene^∗^	Mt hd/2	22.96	119
31	Myrcene	Mt hd/3	23.11	93
32	Pseudocumene	Ar/1	23.11	105
33	2-Pentylfuran	Fur/2	23.22	81
34	(*E,Z*)-2,4-Heptadienal	Ald/11	23.52	81
35	(*Z*)-3-Hexenyl acetate	Est/4	23.53	43
36	Octanal	Ald/12	23.64	84
37	2-Carene	Mt hd/4	23.89	93
38	(*E,E*)-2,4-Heptadienal	Ald/13	24.10	81
39	α-Phellandrene	Mt hd/5	24.15	93
40	α-Terpinene	Mt hd/6	24.50	121
41	*p*-Cymene	Mt hd/7	24.77	119
42	Limonene	Mt hd/8	24.96	68
43	β-Phellandrene^∗^	Mt hd/9	25.15	93
44	Phenylacetaldehyde	Ald/14	25.16	91
45	(*E*)-2-Octenal	Ald/15	25.48	83
46	Salicylaldehyde	Ald/16	25.77	122
47	(*Z*)-Linalool oxide^a,b^	Alc/7	26.34	59
48	Acetophenone	Ket/6	26.38	105
49	(*Z*)-3-Hexenyl propionate	Est/5	26.77	67
50	*(E*)-Linalool oxide^a,b^	Alc/8	26.90	111
51	2-Ethyl hexanoic acid	Acid/3	26.91	88
52	Linalool^a^	Alc/9	27.04	93
53	Nonanal	Ald/17	27.18	57
54	(*Z*)-3-Hexenyl isobutyrate	Est/6	28.25	82
55	Octanoic acid	Acid/4	28.84	60
56	Benzonitrile	Nit/2	28.78	117
57	(*E*)-2-Nonenal	Ald/18	28.92	70
58	Benzyl acetate	Est/7	29.30	108
59	(*Z*)-3-Hexenyl butyrate	Est/8	29.67	67
60	4 Terpineol^a^	Alc/10	30.27	71
61	α-Terpineol^a^	Alc/11	30.65	59
62	Methyl salicylate	Est/9	30.67	65
63	Decanal	Ald/19	31.18	70
64	β-Cyclocitral^c^	Ald/20	31.53	137
65	δ-Elemene^∗^	Sqt/1	34.88	121
66	Eugenol^c^	Alc/12	35.32	164
67	Ethyl decanoate	Est/10	35.76	88
68	α-Ionone^c^	Ket/7	37.25	121
69	β-Caryophyllene	Sqt/2	37.76	133
70	α-Humulene	Sqt/3	38.73	121
71	β-Ionone^c^	Ket/8	38.80	177
72	Methyl dodecanoate	Est/11	39.30	74
73	Nerolidol^d^	Alc/13	40.55	93

*Family Code: Alc, alcohol; Ald, aldehyde; Ar, aromatic hydrocarbon; Est, ester; Fur, furane; Ket, ketone; Mt hd, monoterpene hydrocarbon; Nit, nitrile; Sqt, sesquiterpene. ^∗^Tentative identification based on mass spectrum. ^*a*^Monoterpene-derived compound. ^*b*^In addition to the alcohol group, it has a tetrahydrofuran group. ^*c*^Norcarotenoid compound. ^*d*^Sesquiterpene-derived compound.*

**FIGURE 4:**
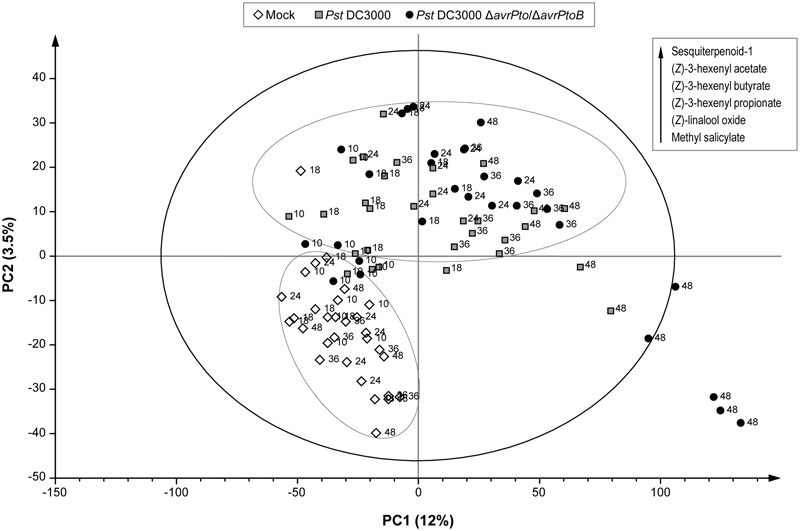
Score plot of the PLS based on the whole array of the mass spectra within a *m/z* range from 35 to 250. (◇) Leaves of the mock-inoculated RG-*Pto* plants, (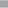) leaves of the RG-*Pto* plants upon infection with *Pst* DC3000, (●) leaves of the RG-*Pto* plants infected with *Pst* DC3000 Δ*avrPto*/Δ*avrPtoB*, at 10, 18, 24, 36, and 48 hpi. Metabolites displayed in the box were identified by loading plot analysis as the responsible for the observed separation.

To manage the large amount of mass data, a multivariate data analysis was performed that consisted in a PLS analysis, where compound abundance was assigned to the X variable, and harvesting time (10, 18, 24, 36, and 48 hpi) and type of infection (mock, compatible, and incompatible interaction) were defined as stepwise Y variables. The PLS analysis (**Figure 4**) showed that the first component (PC1) explained changes in the chemical composition during the experiment (harvesting time), while the metabolic alteration due to bacterial infection was clearly characterized by the second component (PC2). **Figure 4** also displays the over-emitted metabolites in both infected plants, which were identified by the loading plot analysis. As expected, the VOCs from the non-infected plants were chemically similar within 48 h of the experiment. However, the plants infected with both bacterial strains showed an evident variation in their metabolic profile compared to the mock plants. This indicated that VOCs emission was independent of symptomatology. However, no clear separation between the virulent and avirulent infections was observed in this PLS score plot.

**FIGURE 5:**
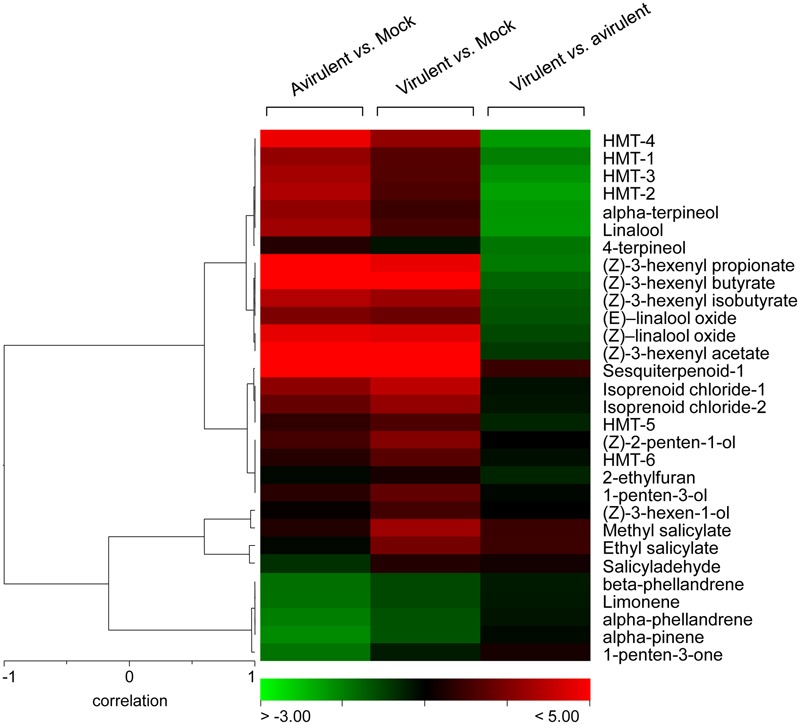
Hierarchical cluster of the volatile compounds from infected plants. Log_2_-transformed ratios are represented as a heat map according to the scale below. Red corresponds to higher values; green denotes lower values. Column 1 represents the ratios of the VOCs emitted by the tomato plants infected with avirulent strain *Pst* DC3000 *versus* the mock-inoculated tomato plants. Column 2 represents the ratios of the VOCs emitted by the tomato plants infected with virulent strain *Pst* DC3000 Δ*avrPto*/Δ*avrPtoB versus* the mock-inoculated tomato plants. Column 3 represents the ratios of the VOCs emitted by the tomato plants infected with virulent strain *Pst* DC3000 Δ*avrPto*/Δ*avrPtoB versus* the tomato plants infected with avirulent strain *Pst* DC3000.

In order to distinguish the defense metabolites associated with each plant–pathogen interaction, the two infections were independently analyzed. The volatile content of the mock-inoculated RG-*Pto* tomato plants was compared with that of the RG-*Pto*-infected one with either avirulent strain *Pst* DC3000 (Supplementary Figure S1) or virulent strain *Pst* DC3000 Δ*avrPto*/Δ*avrPtoB* (Supplementary Figure S2). In these PLS analyses, a marked separation between the infected and mock plants was clearly observed by PC2. In both cases, the loading plot revealed a specific set of VOCs that strongly contributed to the separation of samples according to the specific plant–bacterial infection. **Figure 5** shows a hierarchically clustered heat map, including the most discriminative compounds by comparing the volatile profile of the tomato plants infected with the avirulent or virulent Pst strains with the control plants (column 1 and column 2, respectively).

These defense compounds, induced by both infection types, derive from three important plant metabolic pathways: fatty acids, terpenoids and benzenoids. Among them, the most prominent VOCs produced upon both bacterial infections (red in columns 1 and 2) were some esters of (*Z*)-3-hexenol, such as (*Z*)-3-hexenyl acetate, (*Z*)-3-hexenyl propionate, (*Z*)-3-hexenyl isobutyrate, (*Z*)-3-hexenyl butyrate, some hydroxylated monoterpenes (HMT), such as linalool, α-terpineol, both (*Z*)- and (*E*)- isomers of linalool oxide, and an unidentified sesquiterpene. The statistical analyses showed that their differential induction was significant (Supplementary Table S2). Regarding benzenoids emission, an increase in the production of methyl salicylate (MeSA), salicylaldehyde, and ethyl salicylate was also observed in both infections. The accumulation of the VOCs that derived from salicylate was consistent with the SA accumulation detected in the compatible interaction since levels of these phenolic derivatives were also higher in this virulent infection (**Figure 2**).

### Comparison of the VOCs Profiles of Tomato Leaves upon Infection with Virulent and Avirulent *Pseudomonas syringae* Strains Unraveled a Specific Volatile Response for ETI

**FIGURE 6:**
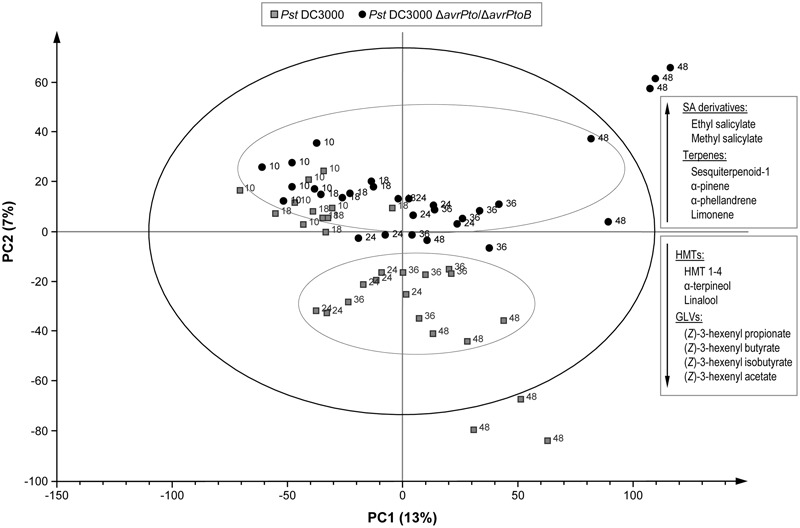
Score plot of the PLS based on the whole array of the mass spectra within a *m/z* range from 35 to 250. (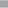) Leaves of the RG-*Pto* plants upon infection *Pst* DC3000, (●) leaves of the RG-*Pto* plants infected with *Pst* DC3000 Δ*avrPto*/Δ*avrPto* at 10, 18, 24, 36, and 48 hpi. Metabolites displayed in the boxes were identified by loading plot analysis as the responsible for the observed separation.

In order to identify whether a specific set of volatile metabolites was involved in the establishment of effective defense such as ETI, another PLS analysis was performed by comparing the VOCs emitted from the tomato plants infected with avirulent strain *Pst* DC3000 and with virulent strain *Pst* DC3000 Δ*avrPto*/Δ*avrPtoB* (**Figure 6**). Once again, the second component clearly showed the different set of volatile compounds emitted by the plant that underwent either a compatible or an incompatible interaction during the 48 hpi period. This metabolomic approach allowed us to identify the differentially induced VOCs in each infection type, by using the loading plot analysis. The hierarchically clustered heat map shows the VOCs that were differentially emitted by the tomato plants infected with virulent Pst strains compared with the avirulent infection (**Figure 5**, column 3). Most VOCs, which were over-emitted by tomato plants during the establishment of the ETI triggered by the avirulent strain (green-colored), showed significant differences compared with the symptomatic infection (Supplementary Table S2).

The specific VOCs differentially released from the symptomatic tomato plants (virulent/avirulent ratio > 1; Supplementary Table S2) were an unidentified sesquiterpene, SA derivatives methyl salicylate and salicylaldehyde, monoterpenes α-pinene, α-phellandrene, β-phellandrene and limonene, as well as two isoprenoid chlorides. The strong induction of the SA derivatives was previously observed when comparing the volatile profiles of these susceptible plants to their corresponding mock-inoculated plants (Supplementary Figure S2). This study also revealed enhanced emission of monoterpenes after infection with virulent strain *Pst* DC3000 Δ*avrPto*/Δ*avrPtoB*.

Interestingly, we identified a set of volatiles that were significantly over-emitted by tomato plants when effectively resisting disease (green-colored in column 3 of **Figure 5**). Among them, several HMT, such as linalool, α-terpineol, 4-terpineol, (*Z*) and (*E*)-linalool oxides, HMT-1, HMT-2, HMT-3, and HMT-4, as well as the esters (*Z*)-3-hexenyl propionate, (*Z*)-3-hexenyl butyrate and (*Z*)-3-hexenyl isobutyrate, were found. The induction of these compounds is mentioned above (when comparing the volatile profiles of the resistant plants with their corresponding mock-inoculated plants; Supplementary Figure S1). The specific over-emission of these green leaf volatiles (GLVs) esters and HMTs during ETI suggests that these VOCs could participate in the defense response.

As a result of these untargeted metabolomic analyses, we conclude that the infected tomato plants emitted quantitatively different volatiles depending on each type of bacterial strain used in this experiment. Monoterpenes and SA derivatives were released at higher rates by the symptomatic plants upon successful bacterial infection, while HMT and hexenyl esters were differentially over-emitted during ETI establishment, which led to resistance.

### Bacterial Infection Induces the Specific Expression of the Genes Involved in VOCs Biosynthesis

**FIGURE 7:**
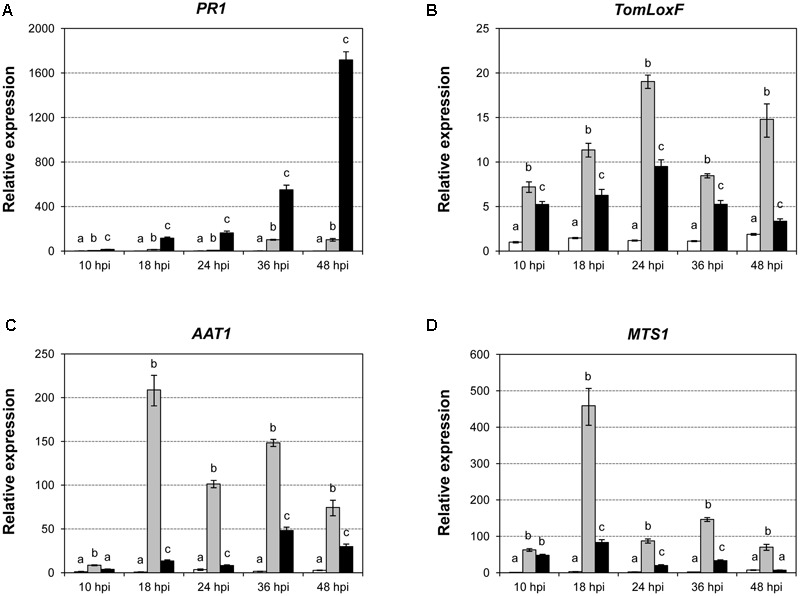
Expression levels of the tomato *PR1* **(A)**, *TomLOXF* **(B)**, *AAT* **(C)**, and *MTS1* **(D)** genes in the mock-inoculated RG-*Pto* tomato plants (white bars) and upon infection with *Pst* DC3000 (light gray bars) or *Pst* DC3000 Δ*avrPto*/Δ*avrPtoB* (dark gray bars) at 10, 18, 24, 36, and 48 hpi, determined by a real-time qRT-PCR analysis. Values were first normalized to the *Elongation Factor 1 alpha* (*eEF1α*) expression level. Expression levels are represented as mean ± standard error of three biological repetitions. An ANOVA analysis was performed at each time point. Different letters indicate the statistical significance differences with *p*-value < 0.05.

To study whether differential volatile production was due to transcriptional activation, we analyzed the expression levels of several key genes involved in the VOC biosynthesis by qRT-PCR. The results of the mock-inoculated RG-*Pto* tomato plants and the plants infected with *Pst* DC3000 or *Pst* DC3000 Δ*avrPto*/Δ*avrPtoB* at 10, 18, 24, 36, and 48 hpi are shown in **Figure 7**. We used the induction of the tomato Pathogenesis-Related *PR1* gene as a positive control of bacterial infection, and observed a correlation of the expression of this gene and symptom development (**Figure 7A**). GLVs esters are known to be synthesized by 13-lipoxygenases (13-LOX) via 13-hydroperoxides, which are later cleaved by 13-hydroperoxide lyases (13-HPL) into (*Z*)-3-hexenal. This last compound is reduced by alcohol dehydrogenase (ADH). Finally, ester formation is catalyzed by alcohol acyl transferases (AAT) (Scala et al., 2013a).

In tomato, six genes that encode various types of lipoxygenases (TomloxA-F) have been described (Mariutto et al., 2011). *TomloxC, TomloxD*, and *TomloxF* encode 13-LOX lipoxygenases, and are involved in the synthesis of oxylipins, which play an important role in the response to biotic stress. Tomlox D lipoxygenase participates in the synthesis of JA, while Tomlox C and Tomlox F are involved in the biosynthesis of GLVs. As **Figure 7B** shows, a significant induction of *TomloxF* was detected upon bacterial infection with both strains at all the studied time points, and this induction was greater when ETI had been established. Regarding alcohol acyltransferases, five *AAT* genes (SlAAT1 - 5) have been identified in tomato (Goulet et al., 2015). We observed that the induction of *AAT1* followed a similar pattern to that of *TomloxF* (**Figure 7C**). These induction patterns statistically correlated with the emission of the GLV esters (Supplementary Table S3A) in both infections, and became higher during ETI establishment. Therefore, these data are consistent with the VOCs metabolomic analysis, and suggest a possible role of the biosynthesis of GLV esters in plant defense against bacteria.

Terpene synthases (TPS) catalyze the synthesis of mono-, sesqui- and diterpenes, and are responsible for the diversity of the isoprene compounds found in nature (Degenhardt et al., 2009). There are 44 TPS genes in *Solanum lycopersicum*, 29 of which are potentially functional (Falara et al., 2011). By qRT-PCR, we observed a significant induction of *TPS5*, also known as *MTS1* (**Figure 7D**), in the immunized tomato plants, which peaked at 18 hpi. This gene induction correlated with the production of several HMTs, such as linalool or α-terpineol (Supplementary Table S3B).

## Discussion

Tomato VOCs have been associated mainly with either improved fruit quality (Rambla et al., 2014) or the response against herbivores (Wei et al., 2013). Here, we extend this knowledge to the volatile differential emission of tomato leaves infected with virulent or avirulent bacteria. We were particularly interested in studying the contribution of VOCs to the resistance mechanisms presented by tomato leaves, which display no symptoms upon bacterial infection (**Figure 1**).

To better characterize both types of compatible and incompatible interactions, the levels of the different signal molecules, e.g., SA, GA, ET, or JA, were measured at several time points (**Figures 2, 3**). Simple natural phenolics SA and GA are fundamental components of the signal transduction pathway, which triggers defense responses against different invading pathogens in many plant species. However, the biological role of these signals depends on the plant–pathogen system (Métraux and Raskin, 1993; Bellés et al., 1999; Dempsey et al., 1999). SA accumulation is associated with incompatible interactions (Métraux et al., 1990; Rasmussen et al., 1991; Malamy et al., 1992; Silverman et al., 1993; Uknes et al., 1993; Shirasu et al., 1997), while GA is associated mainly with compatible plant–pathogen systems (Bellés et al., 2006; López-Gresa et al., 2010). GA could also constitute a signal molecule that is complementary to SA since exogenous treatments with this compound are able to induce different defense responses to those triggered by SA (Bellés et al., 1999; Campos et al., 2014). Plants also produce ET in response to most biotic and abiotic stresses (Abeles et al., 1992; Merchante et al., 2013). ET has been particularly involved in the response of Rutgers tomato plants to bacterial pathogen *Pseudomonas syringae* when infiltrated into leaves (Bellés et al., 1989; Zacarés et al., 2007). Finally, lipid-derived hormone JA has been described as being associated with wounding response and necrotrophic infections, and produces an antagonistic effect on SA-mediated signaling (Zhang et al., 2017). Indeed JA-insensitive tomato *jai1* mutants are more resistant to virulent *Pseudomonas syringae* DC3000 (Zhao et al., 2003). However, jasmonate-deficient *def1* tomato mutants have been described as being more susceptible to *Pseudomonas syringae* and *Xanthomonas campestris* (Thaler et al., 2004), which thus indicates that the role of JA in tomato bacterial infections is still unclear.

We generally observed that SA, GA, and ET accumulated at higher levels in the tomato plants infected with the virulent bacterial strain compared to those infected with avirulent bacteria. These higher levels correlated with symptom severity. SA accumulation has been described to occur at 4 or 10 dpi in tomato plants inoculated with either avirulent or virulent *Xanthomonas*, respectively (O’Donnell et al., 2001; Block et al., 2005). Besides in these tomato interactions, an earlier increase in ET has been described in avirulent infection, while delayed ET synthesis happens in the tomato plants infected with virulent *Xanthomonas* (Ciardi et al., 2000; Block et al., 2005). These results contrast with those observed herein, where the highest ET and SA levels were detected in symptomatic bacterial infection. Several factors could be behind the different SA and ET levels described in each pathosystem, such as timing during infection progress, pathogen dose, greenhouse conditions, or the plant growth stage. Yet in all the cases, the accumulation of either SA or ET correlated with PR1 induction (**Figure 7A**), a classical marker gene that is useful for assessing disease development. The higher level of both ET and GA, detected in the RG tomato plants infected with the virulent *Pst* strain, was associated mainly with symptom development, and agrees with those previously described (Lund et al., 1998; Bellés et al., 2006). Regarding ET, the mutants and tomato genotypes impaired in ET perception or ET synthesis exhibit a significant reduction in disease symptoms *versus* the wild type upon infection with different pathogens (Lund et al., 1998). These previous results present compelling evidence that both biosynthesis and ET perception are critical for symptoms development in tomato leaves.

Unlike the accumulations of SA, GA, and ET, we observed that JA levels drastically lowered in the virulent infection in accordance with the well-known SA–JA antagonism (Zhang et al., 2017). However, in the plants infected with avirulent bacteria, JA accumulation remained comparable to the mock-inoculated plants, similarly to the detected SA levels. Very few reported studies have monitored JA levels in bacteria-infected plants. In Arabidopsis plants, no differences in JA levels between mock-inoculated plants and those infected with a virulent bacteria have been described at 2 and 24 hpi (Scala et al., 2013b). Unlike our dipping infection method, these authors performed inoculation with a syringe, which thus caused mechanical damage to both mock and infected leaves. This difference in the inoculation procedure could explain the divergence of our results with those previously published.

All these data indicate that the different accumulation patterns of these four signal molecules depend on the diversity of pathogens with a range of lifestyles. To our knowledge, this is the first study in which the levels of all these signal molecules have been measured in RG tomato plants infected with both *Pst* strains.

Interestingly, we observed that changes in VOCs emission were mostly independent of macroscopic symptoms since the tomato leaves infected with the avirulent strain overproduced some specific VOCs. We identified 73 emitted VOCs by the GC-MS technology. Although more than 300 VOCs have been reported in tomato fruit (Tikunov et al., 2005), very little information is available on the detailed volatile profile in tomato leaves (Buttery et al., 1987; Wang et al., 2001; Thelen et al., 2005; Zhang et al., 2008; Zhang and Chen, 2009; Proffit et al., 2011), thus our data contribute to this knowledge. The comparison of the VOCs profiles of tomato leaves upon infection with virulent and avirulent *Pseudomonas syringae* strains allowed us to identify the differentially emitted compounds associated with each interaction. The VOCs emission of a diseased leaf is enriched in monoterpenes and SA derivatives, while that of a resisting leaf is characterized mainly by esters of hexenyl GLVs and HMTs.

For the VOCs emitted by symptomatic tomato leaves, the induction of monoterpenes in tomato plants upon *Botrytis* infection has been described, where α-phellandrene and β-phellandrene, 2-carene, limonene, and α-pinene contributed to more than 95% of volatile emissions (Jansen et al., 2009). Nevertheless, the specific release of monoterpenes has been described in pepper plants exposed to the incompatible *Xanthomona*s pathogen, but not in compatible interaction (Cardoza and Tumlinson, 2006). MeSA is generally induced upon pathogen infection (Loake and Grant, 2007). In pepper plants infected with avirulent and virulent *Xanthomonas* strains, MeSA is also emitted at higher levels in the compatible interaction (Cardoza and Tumlinson, 2006). However, in tobacco plants infected with several *P. syringae* strains, MeSA levels become higher upon avirulent inoculation (Huang et al., 2003).

Biotic stresses have been described to trigger emissions of volatile molecules, which are products of the lipoxygenase (LOX) pathway, such as C_6_ aldehydes, alcohols and derivatives, generally referred to as GLVs (Niinemets et al., 2013). For example, *Pst* infection provokes the emission of (*Z*)-3-hexenol and (*E*)-2-hexenal in bean and tobacco leaves, respectively (Croft et al., 1993; Heiden et al., 2003). GLVs are also emitted after fungal and virus infections (Scala et al., 2013a). In tomato plants, (*Z*)-3-hexenol, (*Z*)-3-hexenal, and (*Z*)-3-hexenyl acetate are the dominant LOX products in the volatile emission after *Botrytis cinerea* inoculation (Jansen et al., 2009). Volatile esters not only contribute to the aroma of many fruits and flowers, but are also related to plant defense and plant-to-plant signaling (Goulet et al., 2015). The ester (*Z*)-3-hexenyl acetate is one of the most abundant volatiles to be emitted from mechanically or herbivore-damaged *Arabidopsis thaliana* plants (D’Auria et al., 2007), and can prime a defense response in nearby plants (Engelberth et al., 2004; Frost et al., 2008). The emission of other (*Z*)-3-hexenyl esters has also been described in pepper plants upon *Xanthomonas* infection (Cardoza and Tumlinson, 2006). The induction of (*Z*)-3-hexenol and some of its derived esters upon both bacterial infection types in the tomato plants reported herein extend GLVs emission to other plant–pathogen interactions. Our results, together with those in which (*Z*)-3-hexenol induces defense genes in Arabidopsis and maize plants (Bate and Rothstein, 1998; Farag et al., 2005), suggest that this alcohol can act as a signaling molecule involved in plant response. Another short-chain alcohol, 3-pentanol, has been found to trigger induced resistance in Arabidopsis against *Pseudomonas syringae* (Song et al., 2015), and also in pepper against *Xanthomonas axonopodis* pv. *vesicatoria* (Choi et al., 2014), by priming the SA and JA signaling pathways. Besides, several reports have shown the antifungal (Vaughn et al., 1993) and antibacterial properties (Croft et al., 1993; Deng et al., 1993) of GLVs, which thus reinforces the role of these VOCs in plant defense. Our results also reveal the possible defensive role of GLV esterification since these GLV esters were overproduced during ETI establishment.

Some of these GLVs, including hexanal, (*Z*)-3-hexenal or (*E*)-2-hexenal, have also been described as major compounds emitted by tomato fruit (Rambla et al., 2014), where *Pseudomonas syringae* can cause damage (Xin and He, 2013). Although esterase activity in fruit is enhanced in the red-fruited species of the tomato clade (Goulet et al., 2012), it would be interesting to study whether infection with virulent or avirulent bacteria could produce the esterification of these aldehydes in fruit, which would thus extend the defensive role of these GLVs esters to other organs.

Other VOCs involved in the defense response of plants are terpenoids, which are emitted after wounding or egg deposition by insects. Terpenoids induced by herbivores act in plant defense by attracting insect predators, and by acting as repellents or toxic compounds (Turlings and Tumlinson, 1992; Wegener et al., 2001). Besides, they have been associated to resistance against downy mildew in grapevine (Alarcón et al., 2015). However, the role of terpenoids in plant-bacteria interactions is not well studied. Here, we detected different HMTs, such as (*Z*)- and (*E*)-linalool oxides, linalool, α-terpineol, 4-terpineol, and six putative HMTs emitted from both infected plant types, where induction was greater in avirulent infection. The release of linalool and β-ocimene has also been described in tobacco plants infected by an avirulent strain of *Pseudomonas* (Huang et al., 2003). Since monoterpenes are emitted mainly by symptomatic plants, and HMTs are differentially released by those displaying the immune response, terpene hydroxylation appears to be a key process in the plant defense response.

In order to study whether the increase in VOC was associated with the induction of the VOC biosynthesis machinery, the expression of several genes, e.g., *Tomlox* and *AAT*, involved in the biosynthetic pathway of the esters of GLVs, and *TPS*, implicated in the biosynthesis of terpenoids, was studied by qRT-PCR. We observed a positive correlation in the induction of *TomloxF, AAT1*, and *MTS1* with the emission of the corresponding VOCs, which were differentially released in the tomato plants that displayed ETI.

Among the six described Tomlox isoforms, the induction of the *TomloxF* gene has also been described to result from the infection caused by *Pseudomonas putida* (Mariutto et al., 2011). Our results reinforce the defensive role of this tomato LOX isoform and validate the metabolomic analysis. Regarding the different isoforms of the tomato alcohol acyltransferases, AAT1 has been correlated to the production of GLV esters in tomato fruits (Goulet et al., 2015). Accordingly, we observed the induction of the *AAT1* gene upon bacterial infection, and the corresponding GLV esters accumulation, being this induction greater in the tomato plants that exhibited ETI.

Volatile isoprenoids represent the most abundant group of volatile compounds in plants, and are common components of both their aroma and defensive response induced by herbivores and pathogens (Aharoni et al., 2005). The observed induction of *MTS1* upon bacterial infection correlates with the detected emission of monoterpenes and HMTs, and agrees with the previously described role of TPS in plant defense. Transgenic plants of *Arabidopsis thaliana*, which overexpress the *TPS (E)-β-caryophyllene synthase* gene, emit larger amounts of this sesquiterpene and are more resistant to bacterial infection, which confirms the role of this gene in defense (Huang et al., 2012). *MTS1* expression in tomato leaves has been described to be induced by spider mite-infestation, wounding and JA treatment (van Schie et al., 2007).

Terpene synthases reaction products are subsequently modified by hydroxylation, methylation, acylation, reduction, oxidation, isomerization, or glycosylation, which gives rise to more complex terpene compounds. Hydroxylation of monoterpenes, which would lead to compounds such as α-terpineol, is performed by Cytochrome P450 enzymes (CYP450) (Höfer et al., 2014). There are approximately 250 CYPs in tomato^1^, which means that clarifying which CYP450 might be responsible for terpene hydroxylation and is, therefore, involved in defense through this signaling pathway, is a complex task. However, approximately 25 CYP proteins have been reported to be involved in the immune response of tomato plants to bacteria *Pst*, which helps limit the search of the CYP450 responsible for the hydroxylation of terpenes and confirms the importance of hydroxylation in plant defense response (Pombo et al., 2014).

Our results suggested that the esters of GLVs and HMTs could play a defensive role in the tomato plant response. Unlike SA accumulation, which is the classical signal molecule in incompatible interactions that accumulates at higher levels in virulent infections, these VOCs were differentially emitted at higher levels when plants efficiently resisted bacterial infection, which indicates that they could play a defensive role. Further studies, such as pharmacological or genetic approaches, could be conducted to test this possibility. These volatile compounds could also display interesting biological properties, such as antioxidant, antimicrobial, insecticide or resistance inducers, and could be good candidates for agrochemical and pharmaceutical industries. Besides, the generation of tomato transgenic plants over-expressing enzymes involved in the biosynthesis of these volatiles could result in a new biotechnological strategy to obtain resistance.

## Author Contributions

MPL-G and JMB conceived and designed the study. MPL-G, PL, and LC carried out the experiments. IR prepared the figures. MPL-G, JLR, and AG performed the GC-MS-based metabolomics approach. MPL-G and PL did the data processing and statistical analysis. MPL-G, PL, IR, VC, and JMB interpreted the results. MPL-G and PL wrote the manuscript. JMB handled the literature.

## Conflict of Interest Statement

The authors declare that the research was conducted in the absence of any commercial or financial relationships that could be construed as a potential conflict of interest.

## Footnotes

1 www.solgenomics.net
