# Supplementary material for: A Non-targeted Metabolomics Approach Unravels the VOCs Associated with the Tomato Immune Response against Pseudomonas syringae
Source: Front Plant Sci. 2017 Jul 4;8:1188. doi: 10.3389/fpls.2017.01188 (PMC5495837; doi:10.3389/fpls.2017.01188)
Supplement: Supplementary file 1 [file Table_1.PDF]

Table S1

Gene	Forward primer (5'-3')	Reverse primer (5'-3')
AAT1	TTGCCTGTATGGGAAAGACA	AGATTCCCACGCAATTTTTG
MTS1	TGGTGGTCACCTTCAAGAGA	GCCTTGTGGTGGAAATAGGA
PR-1	ACTCAAGTAGTCTGGCGCAACTC	AGTAAGGACGTTGTCCGATCCAGT
eEF1α	CCACCTCGAGATCCTAATG	ACCCTCACGTATGCTTCCAG

Table S1. Primer sequences used for quantitative RT-PCR analysis of tomato *AAT1*, *MTS1*, *PR-1* and *eEF1α* genes.
