# Supplementary material for: A Non-targeted Metabolomics Approach Unravels the VOCs Associated with the Tomato Immune Response against Pseudomonas syringae
Source: Front Plant Sci. 2017 Jul 4;8:1188. doi: 10.3389/fpls.2017.01188 (PMC5495837; doi:10.3389/fpls.2017.01188)
Supplement: Supplementary file 2 [file Table_2.PDF]

| Class | Volatile Organic Compound | Retention time (min) | Specific ion | Avirulent/Mock 24 hpi | | Virulent/Mock 24 hpi | | Virulent/Avirulent 24 hpi | |
|---|---|---|---|---|---|---|---|---|---|
| | | | | Ratio | Significance | Ratio | Significance | Ratio | Significance |
| Fatty acid derivative | 1-penten-3-ol | 11.17 | 57 | 3.1[a] | $2.3\cdot10^{-7}$ | 5.8[a] | $5.3\cdot10^{-10}$ | 1.9 | 0.057 |
| | 1-penten-3-one | 11.29 | 55 | 0.6 | 0.066 | 1.5[a] | 0.045 | 2.6 | 0.784 |
| | 2-ethylfuran | 11.85 | 59 | 1.9[a] | $2.7\cdot10^{-10}$ | 2.6[a] | $4.9\cdot10^{-10}$ | 1.4 | 0.398 |
| | (Z)-2-penten-1-ol | 14.48 | 41 | 4.2[a] | $1.4\cdot10^{-6}$ | 8.4[a] | $3.9\cdot10^{-10}$ | 2.0 | 0.052 |
| | (Z)-3-hexen1-ol | 18.01 | 67 | 2.1[a] | $5.0\cdot10^{-5}$ | 4.2[a] | $3.1\cdot10^{-6}$ | 2.0 | 0.522 |
| | (Z)-3-hexenyl acetate | 23.53 | 43 | 87.8[a] | $6.7\cdot10^{-25}$ | 94.9[a] | $3.9\cdot10^{-23}$ | 1.1 | 0.098 |
| | (Z)-3-hexenyl propionate | 26.77 | 67 | 46.6[a] | $9.4\cdot10^{-21}$ | 24.9[a] | $4.6\cdot10^{-17}$ | 0.5[a] | 0.003 |
| | (Z)-3-hexenyl isobutyrate | 28.25 | 82 | 14.0[a] | $8.0\cdot10^{-13}$ | 10.8[a] | $6.5\cdot10^{-8}$ | 0.8[a] | 0.020 |
| | (Z)-3-hexenyl butyrate | 29.67 | 67 | 86.9[a] | $3.7\cdot10^{-21}$ | 58.9[a] | $9.6\cdot10^{-20}$ | 0.7[a] | 0.011 |
| Terpenoid | Isoprenoid chloride 1* | 11.72 | 41 | 9.3[a] | $6.0\cdot10^{-9}$ | 15.6[a] | $3.9\cdot10^{-12}$ | 1.7[a] | 0.043 |
| | Isoprenoid chloride 2* | 14.23 | 75 | 6.1[a] | $1.1\cdot10^{-9}$ | 9.8[a] | $3.6\cdot10^{-12}$ | 1.6[a] | 0.041 |
| | α-pinene | 21.51 | 93 | 0.4 | 0.045 | 0.8 | 0.325 | 1.8[a] | $3.2\cdot10^{-3}$ |
| | HMT-1* | 22.72 | 139 | 10.0[a] | $7.7\cdot10^{-9}$ | 5.0[a] | $3.8\cdot10^{-7}$ | 0.5[a] | 0.027 |
| | α-phellandrene | 24.15 | 93 | 0.5 | 0.244 | 0.8 | 0.393 | 1.6[a] | 0.045 |
| | limonene | 24.96 | 68 | 0.6 | 0.063 | 0.9 | 0.063 | 1.6[a] | 0.014 |
| | β-phellandrene* | 25.15 | 103 | 0.6 | 0.396 | 0.9 | 0.083 | 1.5[a] | $7.7\cdot10^{-3}$ |
| | (Z)–linalool oxide | 26.34 | 43 | 24.5[a] | $9.8\cdot10^{-10}$ | 22.8[a] | $1.0\cdot10^{-13}$ | 0.9 | 0.642 |
| | (E)– linalool oxide | 26.90 | 111 | 7.8[a] | $2.8\cdot10^{-11}$ | 6.4[a] | $9.9\cdot10^{-12}$ | 0.8 | 0.625 |
| | linalool | 27.04 | 93 | 11.3[a] | $7.5\cdot10^{-11}$ | 4.4[a] | $3.2\cdot10^{-10}$ | 0.4[a] | 0.016 |
| | HMT-2* | 27.69 | 93 | 13.4[a] | $1.0\cdot10^{-8}$ | 4.7[a] | $8.1\cdot10^{-6}$ | 0.3[a] | $5.18\cdot10^{-3}$ |
| | HMT-3* | 28.73 | 94 | 11.9[a] | $6.0\cdot10^{-9}$ | 4.9[a] | $3.5\cdot10^{-6}$ | 0.4[a] | 0.035 |
| | HMT-4* | 29.22 | 92 | 26.4[a] | $4.8\cdot10^{-10}$ | 9.9[a] | $2.5\cdot10^{-7}$ | 0.4[a] | 0.015 |
| | 4-terpineol | 30.27 | 71 | 3.0[a] | $5.7\cdot10^{-7}$ | 1.7[a] | $4.1\cdot10^{-5}$ | 0.6[a] | $9.6\cdot10^{-3}$ |
| | α-terpineol | 30.65 | 59 | 9.6[a] | $2.6\cdot10^{-10}$ | 3.8[a] | $6.2\cdot10^{-9}$ | 0.4[a] | $8.8\cdot10^{-3}$ |
| | HMT-5* | 31.42 | 93 | 3.4[a] | $1.5\cdot10^{-7}$ | 4.6[a] | $1.9\cdot10^{-10}$ | 1.4 | 0.303 |
| | HMT-6* | 31.49 | 65 | 3.0[a] | $1.3\cdot10^{-4}$ | 5.1[a] | $1.2\cdot10^{-3}$ | 1.7 | 0.642 |
| | Sesquiterpenoid-1* | 40.75 | 81 | 43.8[a] | $1.4\cdot10^{-34}$ | 163[a] | $1.3\cdot10^{-26}$ | 3.7[a] | $3.6\cdot10^{-3}$ |
| Benzenoid | Salicylatealdehyde* | 25.77 | 87 | 1.2 | 0.993 | 2.9[a] | $2.5\cdot10^{-6}$ | 2.5[a] | $7.8\cdot10^{-6}$ |
| | Methyl salicylate | 30.68 | 65 | 2.9[a] | $2.0\cdot10^{-4}$ | 10.9[a] | $2.0\cdot10^{-8}$ | 3.8[a] | $4.0\cdot10^{-3}$ |
| | Ethyl salicylate* | 32.92 | 120 | 1.9 | 0.089 | 7.1[a] | $2.7\cdot10^{-4}$ | 3.8 | 0.186 |

* Tentative identification based on mass spectrum.
[a] Significant differences were shown between the compared groups with $p$ value < 0.01 (Student's *t*-test)
HMT means hydroxylated monoterpenoid

**Table S2**. List of induced VOCs in tomato infected leaves upon infection with *Pst* DC3000 or *Pst* DC3000 Δ*avrPto*/Δ*avrPtoB.* The ratio of induction and Student's *t*-test distribution between the two compared subgroups is shown at 24 hours post-inoculation (hpi).
