# Supplementary material for: A Non-targeted Metabolomics Approach Unravels the VOCs Associated with the Tomato Immune Response against Pseudomonas syringae
Source: Front Plant Sci. 2017 Jul 4;8:1188. doi: 10.3389/fpls.2017.01188 (PMC5495837; doi:10.3389/fpls.2017.01188)
Supplement: Supplementary file 3 [file Table_3.PDF]

A)

Volatile organic compound	*TomLox F*	*AAT*
(*Z*)-3-hexen1-ol	0.701	0.334
(*Z*)-3-hexenyl acetate	**0.923****	**0.644***
(*Z*)-3-hexenyl propionate	**0.940****	**0.624***
(*Z*)-3-hexenyl isobutyrate	0.716	**0.727****
(*Z*)-3-hexenyl butyrate	**0.912***	0.562
1-penten-3-ol	0.751	0.256
1-penten-3-one	0.037	-0.277
(*Z*)-2-penten-1-ol	0.791	0.150
2-ethylfuran	**0.813***	0.570

B)

Volatile organic compound	*MTS1*
Sesquiterpenoid 1	0.324
α-pinene	0.284
α-phellandrene	0.409
limonene	0.362
β-phellandrene	0.461
(*Z*)-linalool oxide	0.420
(*E*)-linalool oxide	**0.608***
linalool	**0.701***
4-terpineol	**0.578***
α-terpineol	**0.661***
HMT-1	0.485
HMT-2	0.119
HMT-3	0.160
HMT-4	0.483
HMT-5	0.402
HMT-6	0.412
Isoprenoid chloride 1	0.067
Isoprenoid chloride 2	0.315

** The correlation is significant at the 0.01 level (2-tailed)
* The correlation is significant at the 0.05 level (2-tailed)

Table S3. Pearson statistical correlations between GLVs **(A)** and terpenoids **(B)** biosynthesis-related gene expression and concentration of the corresponding emitted VOCs.
