# Supplementary figures and images for: A Non-targeted Metabolomics Approach Unravels the VOCs Associated with the Tomato Immune Response against Pseudomonas syringae
Source: Front Plant Sci. 2017 Jul 4;8:1188. doi: 10.3389/fpls.2017.01188 (PMC5495837; doi:10.3389/fpls.2017.01188)

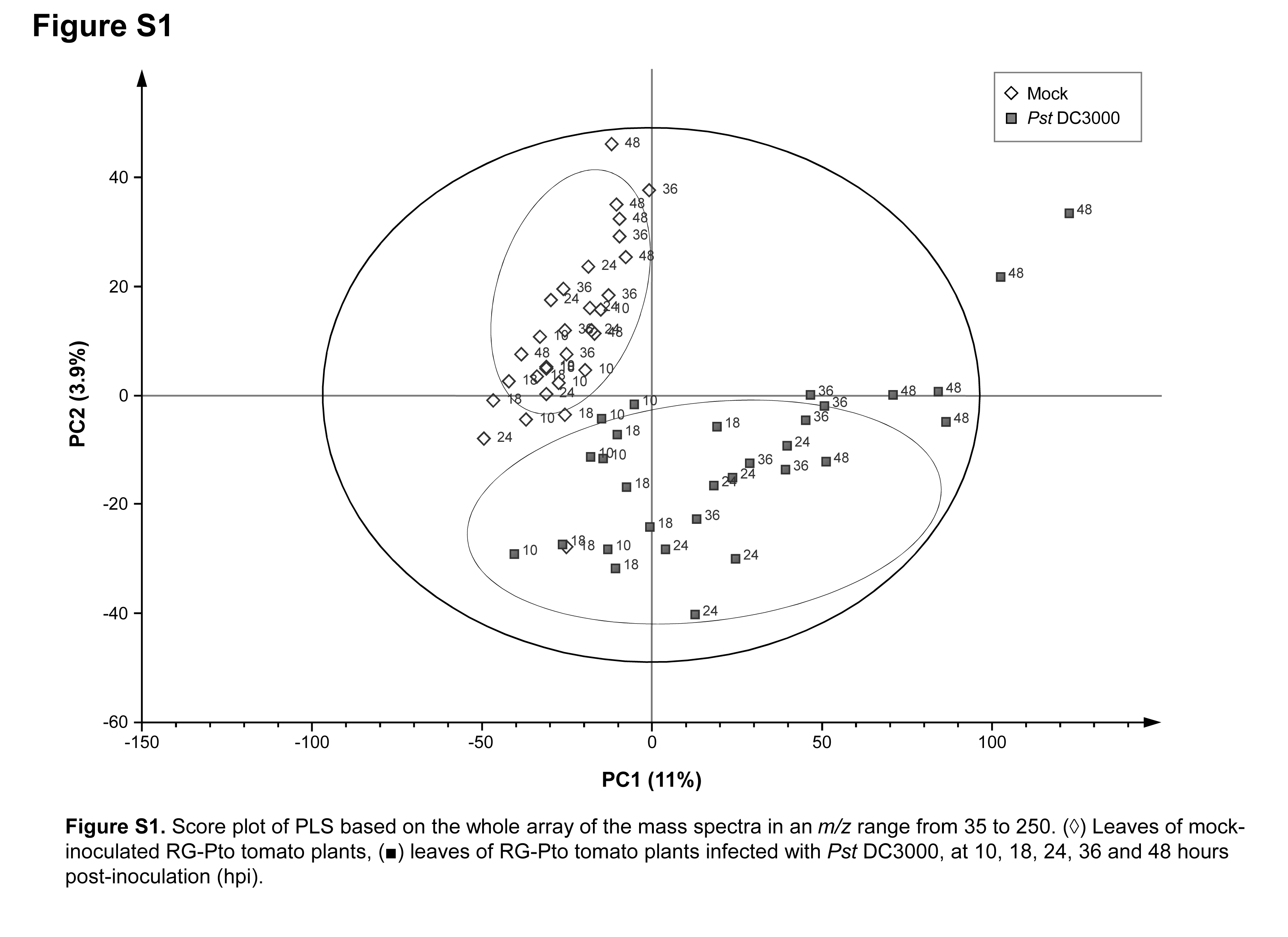

Supplement: Supplementary file 4 [file Image_1.TIF]

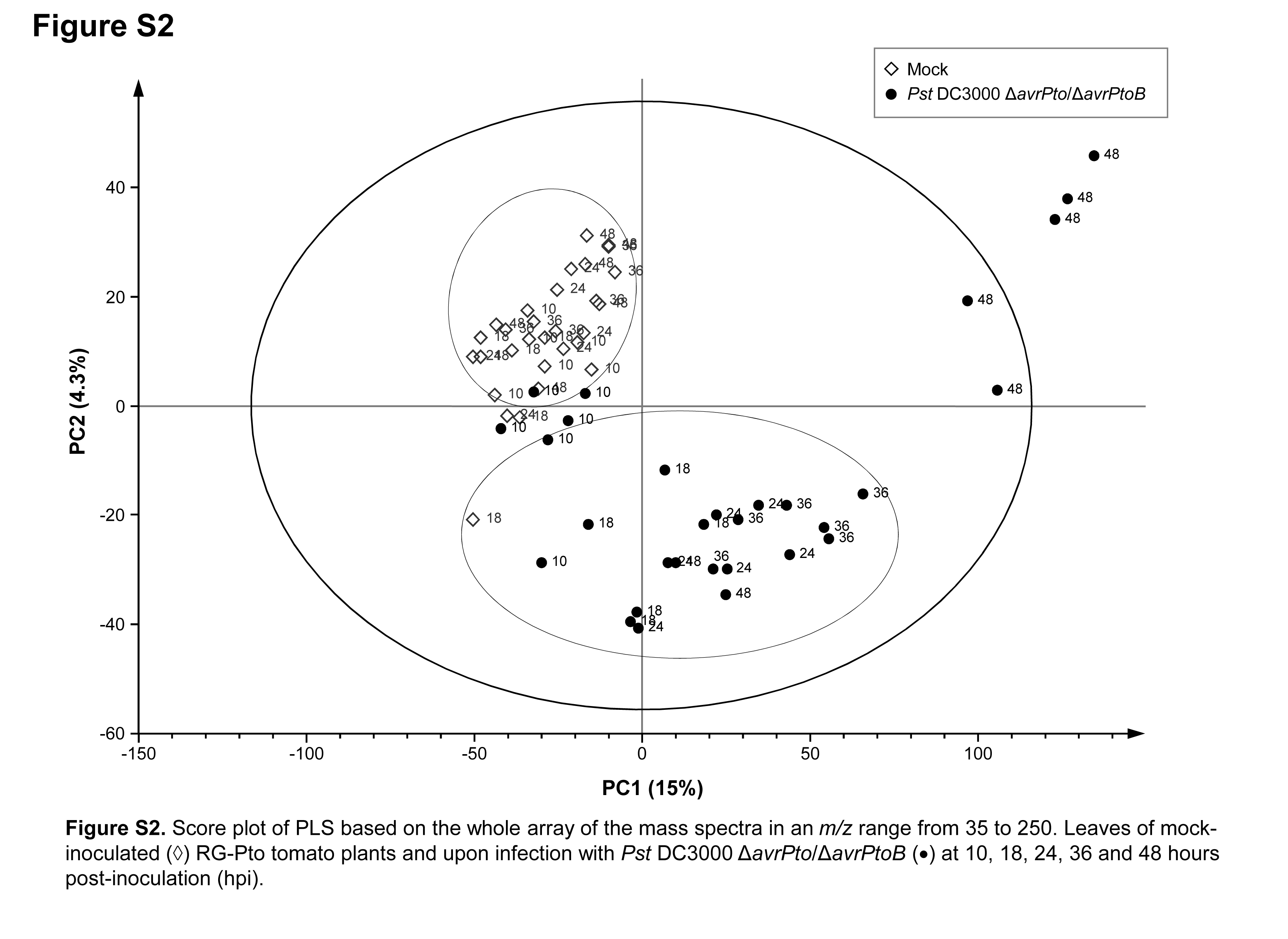

Supplement: Supplementary file 5 [file Image_2.TIF]
